# Pyrophosphate and Irreversibility in Evolution, or why PP_i_ Is Not an Energy Currency and why Nature Chose Triphosphates

**DOI:** 10.3389/fmicb.2021.759359

**Published:** 2021-10-06

**Authors:** Jessica L. E. Wimmer, Karl Kleinermanns, William F. Martin

**Affiliations:** ^1^Institute for Molecular Evolution, Department of Biology, Heinrich Heine University Duesseldorf, Duesseldorf, Germany; ^2^Institute for Physical Chemistry, Department of Chemistry, Heinrich Heine University Duesseldorf, Duesseldorf, Germany

**Keywords:** energetics, bioenergetics, chemical evolution, origin of life, early evolution, metabolism, kinetics, thermodynamics

## Abstract

The possible evolutionary significance of pyrophosphate (PP_i_) has been discussed since the early 1960s. Lipmann suggested that PP_i_ could have been an ancient currency or a possible environmental source of metabolic energy at origins, while Kornberg proposed that PP_i_ vectorializes metabolism because ubiquitous pyrophosphatases render PP_i_ forming reactions kinetically irreversible. To test those ideas, we investigated the reactions that consume phosphoanhydride bonds among the 402 reactions of the universal biosynthetic core that generates amino acids, nucleotides, and cofactors from H_2_, CO_2_, and NH_3_. We find that 36% of the core’s phosphoanhydride hydrolyzing reactions generate PP_i_, while no reactions use PP_i_ as an energy currency. The polymerization reactions that generate ~80% of cell mass – protein, RNA, and DNA synthesis – all generate PP_i_, while none use PP_i_ as an energy source. In typical prokaryotic cells, aminoacyl tRNA synthetases (AARS) underlie ~80% of PP_i_ production. We show that the irreversibility of the AARS reaction is a kinetic, not a thermodynamic effect. The data indicate that PP_i_ is not an ancient energy currency and probably never was. Instead, PP_i_ hydrolysis is an ancient mechanism that imparts irreversibility, as Kornberg suggested, functioning like a ratchet’s pawl to vectorialize the life process toward growth. The two anhydride bonds in nucleoside triphosphates offer ATP-cleaving enzymes an option to impart either thermodynamic control (P_i_ formation) or kinetic control (PP_i_ formation) upon reactions. This dual capacity explains why nature chose the triphosphate moiety of ATP as biochemistry’s universal energy currency.

## Introduction

Starting in the 1960s, thoughts on the possible evolutionary significance of inorganic pyrophosphate (PP_i_) have centered around two main concepts: irreversibility and energy. Kornberg, who worked on nucleic acid polymerization, recognized that PP_i_ producing biochemical steps confer the property of irreversibility upon reactions under physiological conditions because ubiquitous pyrophosphatases constantly degrade PP_i_ in the cytosol of cells (Kornberg, 1962). His reasoning was straightforward: By degrading PP_i_, a substrate required for the enzymatic back reaction of the PP_i_ producing step, the rate of the back reaction effectively approaches zero. In this way, pyrophosphatases would render PP_i_ producing reactions irreversible by means of kinetics, rather than thermodynamics. Though Kornberg’s mechanism of irreversibility was later called into question because PP_i_ concentrations in exponentially growing cells were reported to be too high for this principle to work (Kukko and Heinonen, 1982), as soon as cells leave the exponential growth phase, Kornberg’s principle immediately applies, as we will see during the course of this paper, because PP_i_ production is strictly linked to growth, while PP_i_ hydrolysis is not. Kornberg’s list of such irreversible PP_i_ producing reactions included nucleic acid polymerization, translation, and cofactor biosynthetic routes (Kornberg, 1962) and this function, irreversibility, was seen as harboring the significance of PP_i_.

Lipmann, who worked on high energy bonds, suggested that PP_i_ could have served as a possible energy currency in primordial metabolism, and that modern PP_i_-dependent enzymes represent fossils from a time in which prebiotic metabolism extracted energy from environmentally available phosphate minerals (Lipmann, 1965). In that view, the evolutionary significance of PP_i_ is sought in its possible role as a source of biochemical energy in prebiotic chemical reactions resembling those of physiology. Aspects of both Kornberg’s and Lipmann’s views are germane to Schramm’s proposal that environmental polyphosphates could have powered early nucleic acid synthesis (Schramm et al., 1962).

In 1966, Baltscheffsky reported a membrane-associated pyrophosphatase (mPPase) that reversibly couples proton translocation to PP_i_ hydrolysis (Baltscheffsky et al., 1966), thereby linking PP_i_ to Mitchell’s then new chemiosmotic theory of ATP synthesis involving ion gradients and electron transfer chains (Mitchell, 1961). That finding, together with Reeves’ report of a PP_i_-dependent glycolytic enzyme (Reeves, 1968), now called pyruvate orthophosphate dikinase, seemed to support an ancient bioenergetic role behind the possible evolutionary significance of PP_i_. Based on such findings, the view that PP_i_’s evolutionary significance resides in primordial energetics established a long tradition that is still widely embraced (de Duve, 1991; Russell and Hall, 1997; Russell et al., 2013, 2014; Wang et al., 2019; Piast et al., 2020) though seldom critically inspected (Martin, 2020).

Comparatively few enzymatic reactions involve PP_i_. Kornberg (1962) listed 35 enzymatic reactions that release PP_i_ in the physiological reaction. Heinonen (2001) listed 173 PP_i_ producing reactions. By contrast, Kyoto Encyclopedia of Genes and Genomes (KEGG) list 194 reactions among prokaryotes that involve ATP. Since the book of Heinonen (2001), some new PP_i_ producing reactions have been reported (Nagata et al., 2018), yet the precise roles of PP_i_ in physiology and evolution are still discussed (Heinonen, 2001; Pérez-Castiñeira et al., 2021). Soluble pyrophosphatases (sPPases) are ubiquitous in distribution (Lahti, 1983). Ion-pumping mPPases are found in various microbes and plants (Serrano et al., 2007), and PP_i_-dependent glycolytic enzymes occur as alternatives of ATP-dependent forms, at the phosphofructokinase (PFK) and pyruvate kinase (PYK) steps (Heinonen, 2001; Siebers and Schönheit, 2005; Bräsen et al., 2014; Holwerda et al., 2020). Though PP_i-_dependent glycolysis is often interpreted as an adaptation that reduces ATP expense (Heinonen, 2001) or that salvages energy from PP_i_ produced from translation (Reeves, 1968), PP_i_-utilizing glycolytic enzymes have a conspicuous tendency to occur among microbes that have specialized to sugar-rich environments. Such specialists include human parasites, such as *Entamoeba* (Reeves, 1984), *Giardia* (Müller et al., 2012), and trypanosomes (Michels et al., 2006), as well as non-parasitic cellulose-, saccharose-, and sugar-degrading bacteria (Bielen et al., 2010; Holwerda et al., 2020) and archaea (Bräsen et al., 2014). In addition, PP_i_-dependent enzymes are particularly common in the strictly sugar-based carbon metabolism of plants (Serrano et al., 2007). This pattern of occurrence might be suggestive of an ecological rather than energetic basis behind the distribution of PP_i_-dependent glycolytic pathways.

In line with that view, the use of PP_i_-dependent glycolytic enzymes generally coincides with loss of allosteric regulation through the pathway (Siebers and Schönheit, 2005; Bräsen et al., 2014). In the well-studied example of trypanosomes, loss of regulation allows flux through the pathway to be governed by sugar concentrations in the medium (blood sugar), an ecological adaptation of growth rates to substrate availability, not energetic efficiency, especially as trypanosomes excrete the energy rich compound pyruvate as a metabolic end product (Michels et al., 2006). Even in the well-studied glucose fermenting bacterium *Clostridium thermocellum*, which also excretes pyruvate, a clear energetic advantage of its PP_i_-dependent glycolysis is not evident (Holwerda et al., 2020). Moreover, deletion of *C. thermocellum*’s mPPase has no impact on growth (Holwerda et al., 2020), a finding that is hard to reconcile with a central role for energy conservation *via* PP_i_ in energy metabolism of the bacterium, although sPPase activity was not reported in the mPPase mutant. By contrast, deletion mutants of sPPases are lethal in *Escherichia coli* (Chen et al., 1990) and in yeast (Pérez-Castiñeira et al., 2002). This finding is very notable because from an energetic standpoint, because sPPases effectively “waste” phosphoanhydride bonds *via* rapid PP_i_ hydrolysis, raising the question: why should elimination of the “energy wasting” reaction catalyzed by sPPase be lethal? The growth inhibiting phenotype of sPPase deletion mutants is, however, readily reconciled with Kornberg’s kinetic view of PP_i_ function, because sPPase knockouts in *E. coli* and yeast yield cells that cannot grow mainly because protein synthesis comes to a halt through product inhibition *via* PP_i_ accumulation at the amino acyl tRNA synthesis step.

Our present interest in PP_i_ stems from comparative physiological investigations into the energetics of primordial metabolism (Martin and Russell, 2007; Sousa et al., 2013; Sousa and Martin, 2014; Preiner et al., 2020). We reasoned that if PP_i_ had played any role in primordial energetics, as is widely assumed (de Duve, 1991; Russell et al., 2013, 2014; Wang et al., 2019; Piast et al., 2020), evidence for that role should be preserved in the conserved core of metabolism within modern cells. This is the same conventional logic that is used to interpret other aspects of physiology as relicts of ancient metabolism: metal sulfide clusters in proteins (Eck and Dayhoff, 1966; Wächtershäuser, 1992; Heinen and Lauwers, 1997), the use of organic cofactors as catalysts (White, 1976), carbon metal bonds in enzyme active sites (Martin, 2019), thioesters as energy currencies (Semenov et al., 2016; Kitadai et al., 2021), or anaerobic chemolithoautotrophy (Mereschkowsky, 1910; Decker et al., 1970). Though this line of reasoning (comparative physiology) can be questioned, it is the same reasoning that underlies the view that PP_i_ is an ancient energy currency. The conserved core of metabolism is a set of roughly 400 reactions that generates the 20 canonical amino acids, the four bases of RNA and DNA, and the cofactors required for their synthesis from H_2_, CO_2_, NH_3_, H_2_S, P_i_, and inorganic salts (Wimmer et al., 2021). Because of its universally conserved nature, this biosynthetic core of chemical reactions (though not necessarily all of it enzymes) was present in the last universal common ancestor, LUCA, and has persisted in all lineages throughout evolution over the last 4 billion years since their divergence from LUCA (Weiss et al., 2016). The universal core thus harbors insights not only into LUCA’s physiology, but also into the primordial set of reactions that gave rise to the building blocks from which LUCA was assembled. Because our present investigation probes metabolism itself, our insights into the role of PP_i_ in evolution differ from those based in the study of phosphorous minerals (Pasek et al., 2017). And because our investigation is based in the comparative physiology of living cells, our insights into the role of thermodynamics and kinetics in evolution differ from those based in studies of chemical nucleic acid synthesis (Pascal et al., 2013). A fresh look at the role of PP_i_ in ancient metabolism suggests that Lipmann was probably wrong, that Kornberg was probably right, and furthermore reveals why nature chose triphosphates as the universal energy currency.

## Materials and Methods

### Reactions Involving Inorganic Pyrophosphate

The 36 metabolic reactions involving inorganic pyrophosphate (PP_i_; Supplementary Table S1) in the biosynthetic core were taken from supplemental data of Wimmer et al. (2021). Reaction R00720 involving IMP synthesis was removed from the core because it is not essential. The reactions were initially collected from the KEGG (Kanehisa and Goto, 2000), version December 2020 and polarized in the direction of cell synthesis.

### ATP Hydrolysis Among Prokaryotes

Reactions involving ATP hydrolysis of the reaction scheme X+ATP ↔ Y+ADP+P_i_ were obtained from KEGG (Kanehisa and Goto, 2000). *X* and *Y* are placeholders for variable compounds. Additional compounds on both sides can be present. A total of 15,339 KEGG reactions were searched for the reaction scheme in the forward and back direction since KEGG reactions are not polarized in general. A total of 131 reactions involving ATP hydrolysis were obtained in the data, whereas 61 reactions are specific to prokaryotes (Supplementary Table S2). The domain check was performed by parsing the Enzyme Commission (EC) numbers of each reaction, gathering a list of genes and their respective organisms leading to the domain.

### Collection of Michaelis–Menten Constants for Pyrophosphatases

Michaelis–Menten constants (*K_m_*) for inorganic pyrophosphatase activity in *E. coli* wildtypes were obtained from BRENDA (Schomburg et al., 2002) *via* EC number 3.6.1.1. *Escherichia coli* mutants were removed from the data (Supplementary Table S3).

### Kinetic Effect of PP_i_ in Translation

To investigate the effect of pyrophosphate hydrolysis on the product yield (adenylated amino acid) in aminoacyl-tRNA synthetase (AARS) reactions more quantitatively, kinetic simulations of substrate binding and activation of isoleucine by adenylation in isoleucyl-tRNA synthetase were performed using Mathcad 2001 (Mathsoft Engineering & Education, Inc.). The underlying kinetic scheme is taken from Pope et al. (1998) with hydrolysis of PP_i_ added (see Supplementary Figure S1A). The rate equations were used to obtain the concentration vs. time profiles by numerical integration (see Supplementary Figure S1B). Experimental rate constants were obtained from Pope et al. (1998) and Stockbridge and Wolfenden (2011). Initial concentrations of 1mM amino acid, enzyme, and ATP were used, and integration was carried out up to 20s. These calculations provide an empirical basis for the intuitive effect of product removal during the PP_i_ forming step of translation (see Supplementary Figure S2).

## Results

### Pyrophosphate Polarized LUCA’s Core Biosynthetic Metabolism

To see whether PP_i_ might have had a role in primordial energetics, the reactions of the core (Wimmer et al., 2021) that involve PP_i_ or ATP were identified and polarized in the biosynthetic direction, that is, from H_2_ and CO_2_ toward cell mass synthesis. In Lipman’s view, PP_i_ was an environmental energy source, a substrate that assumes a thermodynamic role as an educt residing on the left side of an enzymatic reaction, while in Kornberg’s view PP_i_ is synthesized in metabolism *via* ATP hydrolysis and assumes a kinetic role as a product that is removed from the right side of the reaction. Writing the reactions from left to right in the direction of CO_2_ to products as the pathways are mapped in KEGG (Kanehisa and Goto, 2000) brings the role of PP_i_ in the core into focus. Among the 36 reactions of the core in which PP_i_ occurs, it is always a reaction product occurring on the right side of the reaction, serving as an energy source in zero reactions (Supplementary Table S1). The reactions of the biosynthetic core of metabolism thus speak 36:0 in favor of PP_i_ conferring irreversibility, as Kornberg (1962) suggested, and harbor no traces of Lipmann’s proposal for an ancient energetic or thermodynamic role for PP_i_.

In the metabolism of modern cells, PP_i_ is always produced from ATP by reaction sequences that sum to ATP+H_2_O→AMP+PP_i_ (∆*G*_o_ *ʹ*=−46kJ·mol^−1^), slightly more exergonic than ATP+H_2_O→ADP+P_i_ (∆*G*_o_ *ʹ*=−32kJ·mol^−1^). This opens the possibility that PP_i_ formation might have played an energetic role in the core, but not as a source of high energy bonds. Were the role of PP_i_ in the core thermodynamic, it could have readily been replaced in evolution by compounds with a similar or higher free energy of hydrolysis, such as acetyl phosphate (∆*G*_o_ *′*=−43kJ·mol^−1^), 1,3-bisphosphoglycerate (∆*G*_o_ *′*=−52kJ·mol^−1^), or phosphoenolpyruvate (∆*G*_o_ *′*=−62kJ·mol^−1^), the high energy bonds in all three of which are synthesized in metabolism using one ATP each (the same cost as PP_i_ hydrolysis). Because ATP hydrolysis to adenosine monophosphate (AMP) and PP_i_ is not replaced by alternative energy currencies with a higher free energy of hydrolysis, and because PP_i_ is always a product in the core, not an educt, the function of PP_i_ in the core can hardly be thermodynamic.

Keeping in mind that Kornberg’s suggestion for the role of PP_i_ was based on nucleic acid polymerization and translation, the occurrence of PP_i_ in the core solely as a product suggests that its role is kinetic, lowering the rate of back reactions, rather than thermodynamic. Is this true more generally in metabolism, that is, outside the core? We consulted KEGG. If PP_i_ had any role during early evolution as an energy currency, then some reactions should persist in which PP_i_ hydrolysis is coupled to an otherwise endergonic reaction. Though a handful of PP_i_ consuming reactions phosphorylate substrates in the physiological reaction (Nagata et al., 2018), among 15,339 reactions in KEGG, we found no PP_i_ hydrolyzing, non-phosphorylating reactions at all that provide energetic coupling to an otherwise thermodynamically unfavorable reaction. That is, there were no reactions of the type X+PP_i_ ↔ Y+2 P_i_, whereby we note that the pyrophosphatase reaction, KEGG reaction number R00004, employs H_2_O as X but has no Y component. This is a noteworthy observation. It indicates that PP_i_ serves at best as a phosphorylating agent in metabolism, but never as a source of pure thermodynamic impetus to help push unfavorable reactions forward *via* coupling to PP_i_ hydrolysis. By contrast, a number of metabolic reactions (61 prokaryote specific reactions among 15,339 total reactions in KEGG; Supplementary Table S2) go forward because they are coupled to non-phosphorylating ATP hydrolysis in reactions of the type X+ATP ↔ Y+ADP+P_i_. The lack of such reactions for PP_i_ in KEGG clearly indicates that PP_i_ is not a dedicated energy currency in biosynthesis, notwithstanding the existence of PP_i_-dependent glycolytic pathways, as outlined in the introduction. Note that KEGG does not include the myriad reactions in which ATP (or GTP) phosphorylates proteins, and we know of no examples in which PP_i_ is used to phosphorylate proteins as true energy currencies do. These findings indicate that PP_i_ is not a dedicated energy currency and that by inference, in the simplest interpretation, it never has been.

PP_i_ producing reactions are generally seen as being irreversible under physiological conditions because of the ubiquitous presence of high activities of sPPases in cells (Lahti, 1983; Danchin et al., 1984; Heinonen, 2001), which catalyze the reaction PP_i_+H_2_O→2P_i_ (∆*G*_o_ *′*=−21kJ·mol^−1^), thereby continuously removing a substrate for PP_i_ producing reactions in the reverse direction. Notably, aqueous Mg^2+^ ions alone accelerate the rate of spontaneous PP_i_ hydrolysis by three orders of magnitude in water and PP_i_ hydrolysis in dimethyl sulfoxide/water by six orders of magnitude (Stockbridge and Wolfenden, 2011), such that inorganically catalyzed PP_i_ hydrolysis might have been a mechanism of irreversibility even before the advent of enzymes. Irreversibility at 36 PP_i_-dependent enzymatic reactions in the core – nine in amino acid pathways, three in nucleotide synthesis, and 19 in cofactor synthesis (Supplementary Table S1) – functions in modern metabolism as a system of check valves (valves that close to prevent backward flow) that, individually and in concert, act as a ratchet’s pawl, inching the reactions of the core unidirectionally forward toward product synthesis. We suggest that this has been the case since the availability of ATP as the universal energy currency.

### Pyrophosphate Polarized Metabolism *in toto* Throughout All of Evolution

In the metabolism of LUCA, PP_i_ forced the reactions of the core forward in the direction of monomer synthesis for cell mass synthesis. The effect of PP_i_, however, extended well beyond LUCA’s core biosynthesis because PP_i_ renders nucleic acid and protein synthesis irreversible (Kornberg, 1962), and because LUCA possessed the genetic code and was able to synthesize RNA, DNA, and proteins (Weiss et al., 2016). To get a better picture of the polarizing role of PP_i_ in the central dogma of molecular biology, we generated estimates for its quantitative contribution to the overall ATP budget based on the classical estimates of Stouthamer (1978), which are still in wide use today. Protein synthesis requires activated amino acids, rRNA, tRNA, and mRNA. In a modern cell growing from H_2_, CO_2_, and NH_3_, the synthesis of protein comprises the combined energetic cost of making RNA and protein, consuming roughly 76% of the biosynthetic ATP budget (Table 1). The quantitative contribution of PP_i_ forming reactions to the cellular energy budget is surprisingly large. In amino acid biosynthesis, 47% of the ATP consuming reactions (8/17) generate PP_i_, whereas in nucleotide synthesis 13.6% (3/22) generate PP_i_. In polymerization reactions, the contributions are greater.

**Table 1:** ATP expense per gram of cells during biosynthetic processes^a^ [mol·10^4^].

Component	Monomer synthesis^b^		Polymerization^c^			Total	PP_i_- forming	Total	PP_i_- forming
Protein	14	6.6	191	91.3
RNA	34	4.6	23	20.3
DNA	9	1.2	2	1.2
Lipid^d^	1	–	–	–
Polysaccharide^d^	21	–	–	–
Import^e^	52	–	–	–
Sum	131	12.4	216	113

Energetic cost of protein synthesis incl. Ribosome biosynthesis: 262/347=76%

Energetic contribution of PP_i_ forming steps in cell biosynthesis: 125/347=36%

Energetic contribution of GTP-dependent biosynthetic steps^f^: 98.5/347=28%

a Values are for *Escherichia coli* from Stouthamer (1978) as tabulated by Harold (1986). Lever et al. (2015) calculate ∆*G*_o_^’^ for the synthesis of monomers from H_2_, CO_2_, and NH_3_ based on the values of Neidhardt et al. (1990) but not the ATP expense per monomer. Neidhardt et al. (1990) estimate ATP expense for monomer synthesis as 42 ATP per 20 amino acids and 40 ATP per four nucleotides in *E. coli*. A dash (−) indicates that the value is zero or negligible. Note that these calculations entail only biosynthetic costs and do not consider energy spilling (Tempest and Neijssel, 1984) or maintenance energy, which can exceed biosynthesis by a factor of 3 in exponential growth (Russell and Cook, 1995; Russell, 2007), and by more under energy limitation (Lever et al., 2015).

b The proportion of PP_i_ forming steps in monomer biosynthesis was calculated as the total cost for the monomer multiplied by the fraction of PP_i_ forming steps among ATP hydrolyzing steps en route to nucleoside monophosphates (Wimmer et al., 2021).

c The proportion of PP_i_ forming steps in polymerization takes the costs of proofreading, assembly and modification from Lever et al. (2015) into account. These are not PP_i_-forming reactions.

d LPS, lipopolysaccharide. These values are for *E. coli*. There is a PP_i_ forming component of lipid synthesis in archaea that is neglected here. Archaea also lack LPS and possess no murein, though sometimes pseudomurein (Albers and Meyer, 2011), and have a larger protein component in the cell wall (S-Layer).

e Stouthamer (1978) calculates the cost of import for precursors, mainly ammonium, Lever et al. (2015) neglect import. If one considers a functional core before the origin of free-living cells, no costs for import are incurred.

f In the core, 13% of the triphosphate expense for amino acid and NMP/dNMP monomer synthesis (5510^−4^mol ATP per gram of cells) is GTP-dependent (7.210^−4^mol ATP per gram of cells), plus two GTP-dependent steps in translation (91.310^−4^mol ATP per gram of cells) yield *ca.* 98.510^−4^mol GTP per gram of cells.

**Figure 1:**
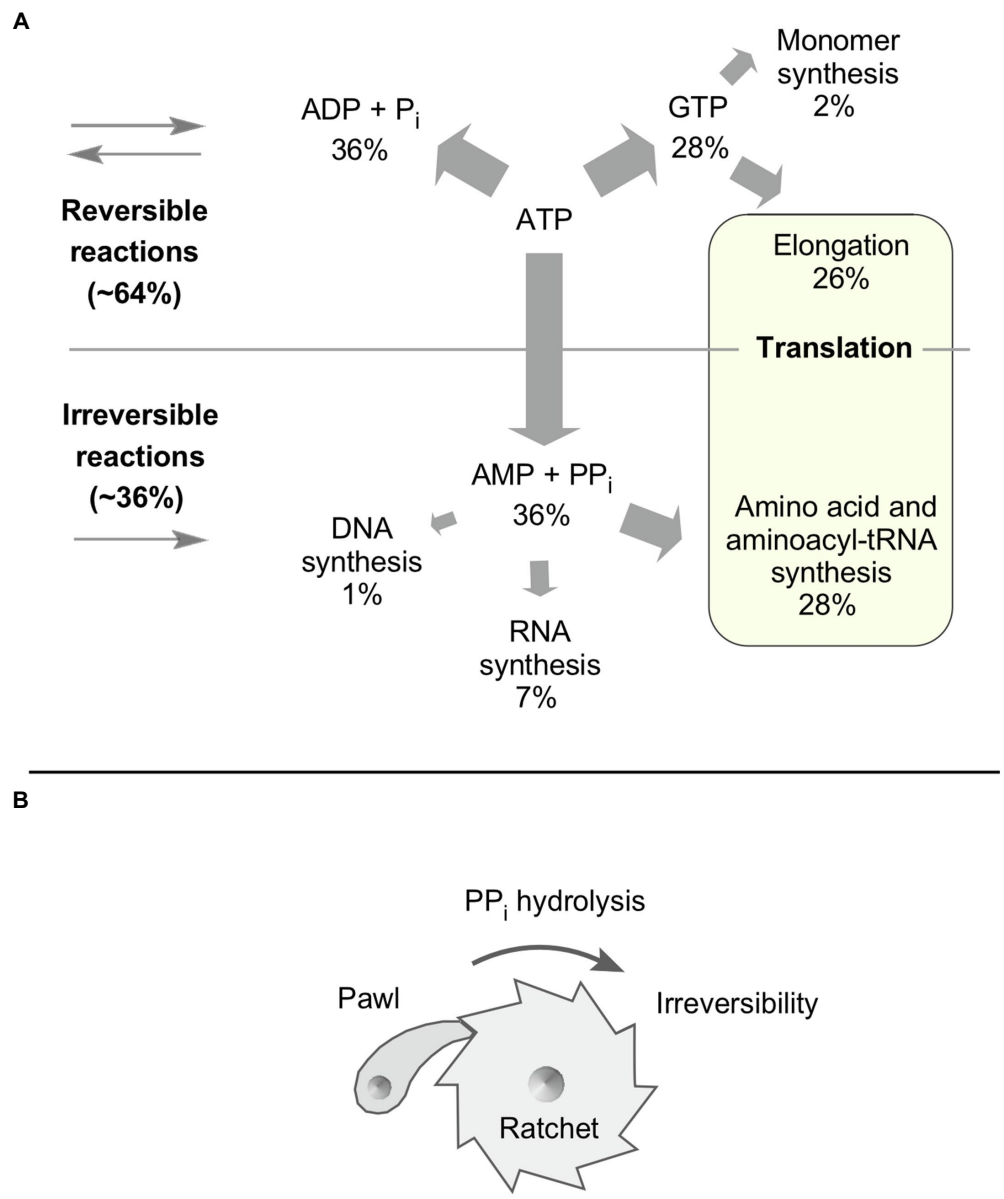
A divide in biosynthetic energy expense. **(A)** Based on Table 1, a summary of triphosphate expenses across biosynthetic processes. **(B)** A ratchet and a ratchet’s pawl as a mechanism of irreversibility. In metabolism, PP_i_ hydrolysis functions as the pawl.

Guanosine triphosphate (GTP) hydrolyzing reactions are not uncommon in LUCA’s biosynthetic core (Figure 1), in line with its ancient role in metabolism (Martin and Russell, 2007) and the observation that in some organisms where it has been investigated, GTP is readily used as a substrate in reactions that are typically regarded as ATP dependent (Holwerda et al., 2020). About 26% of a cell’s energy budget is consumed in the GTP-dependent steps of translation. The main biosynthetic ATP expense in protein synthesis is translation, which consumes four ATP per peptide bond (Stouthamer, 1978). Peptide chain elongation at the ribosome has two P_i_ forming GTP hydrolysis steps catalyzed by EF-Tu and EF-G (Satpati et al., 2014), while amino acyl tRNA synthesis requires the expense of two ATP through amino acid activation by amino acyl tRNA synthetase (AARS) enzymes. Activation generates aminoacyl adenylate and PP_i_, followed by aminoacylation of tRNA and AMP release (Gomez and Ibba, 2020). Because half of the energy cost for translation resides in the PP_i_ producing nature of the AARS reactions, roughly 26% of the cell’s total biosynthetic ATP expense (91/347, Table 1) is incurred to pay the price of irreversibility at the formation of aminoacyl tRNAs for translation.

PP_i_ has an ancient and conserved function in metabolism as a mediator of irreversibility (Kornberg, 1962) that clearly traces to LUCA (Figure 1). By contrast, not a single reaction in the core uncovers a role for PP_i_ as an energy currency in primordial metabolism. Based upon the conserved nature of the core across all modern lineages, we can infer that PP_i_ generating reactions have vectorialized monomer synthesis of ABC compounds throughout evolution, acting as a ratchet’s pawl (Figure 1B), rendering monomer synthesis unidirectional, even in low energy environments.

PP_i_ producing steps in RNA monomer and polymer synthesis account for about 7% of the overall biosynthetic energy budget (Figure 1). As with translation, thermodynamics do not strictly demand PP_i_ generation and hydrolysis, as transcription can operate with NDPs *in vitro* (Gottesman and Mustaev, 2019). PP_i_ production during DNA synthesis only accounts for 1% of the cell’s energy budget (Figure 1), whereby some DNA polymerases can also operate with NDP substrates (Burke and Lupták, 2018). In addition, some polymerases use the irreversible effect of PP_i_ hydrolysis by possession of a pyrophosphatase domain that cleaves PP_i_ as the enzyme moves forward (Kottur and Nair, 2018).

PP_i_ generation and hydrolysis render both the reactions of the core and polymerization reactions during replication, transcription and translation irreversible under the physiological conditions of the cell. In the core, and in the cytosol, this ratchet has locked biochemistry in the direction of cell synthesis during the 4billion years since metabolic origin. The insight of Kornberg (1962, p. 261) “*Hydrolysis of the latter* [pyrophosphate] *by inorganic pyrophosphatases promotes the irreversibility of the synthetic route to coenzymes, nucleic acids, proteins, and structural carbohydrates and lipids*” still stands.

### The Effect of PP_i_ in Translation Is Demonstrably Kinetic

The vectorializing effect of PP_i_ in translation is not thermodynamic, it is the same as in the biosynthetic core: a kinetic ratchet afforded by ubiquitous pyrophosphatases that render protein synthesis unidirectional toward growth. We were able to demonstrate this effect by calculating the kinetics of aminoacyl tRNA synthesis using published rate constants obtained from Pope et al. (1998) and from Stockbridge and Wolfenden (2011). The equations are given in Supplementary Figure S1, into which we introduced values from the literature to obtain the kinetics (the time dependence of reactant and product concentrations) for the isoleucyl-tRNA synthetase reaction in *E. coli* in the presence of inorganic pyrophosphatase. The result is shown in Supplementary Figure S2. The *E. coli* enzyme belongs to the family I or type I PPase, which typically have rate constants on the order of 200–400s^−1^ (Kajander et al., 2013). Using the *E. coli* sPPase I rate constant of 570s^−1^ provided by Stockbridge and Wolfenden (2011) in our calculations, PP_i_ hydrolysis is essentially complete after 0.4s (Supplementary Figure S2). This is a clear result: Pyrophosphatase activity drives the overall reaction of aminoacyl tRNA synthesis forward by removing PP_i_ at a high rate relative to other steps of the reaction such that the adenylated amino acid is formed irreversibly. The reaction kinetics provides a clear empirical basis for the intuitive effect of product removal during the PP_i_ forming step of translation.

Different PPases have, however, different rate constants for PP_i_ hydrolysis. In particular, the membrane bound mPPases are extremely slow; hence, it was of interest to see if they could still provide a similar kinetic effect in the AARS reaction. The mPPases occur in roughly 25% of prokaryotes; the enzyme is also common among protists and is ubiquitous among land plants, where it couples PP_i_ hydrolysis to the pumping of ions (Na^+^ or H^+^) out of the cell or, in the case of vacuolar PPases (vPPases), from the cytosol into vacuoles or acidocalcisomes, organelles rich in calcium and polyphosphate (Kajander et al., 2013). The mPPases and vPPases are one to two orders of magnitude slower than type I sPPases, with rates on the order of 3.5–20s^−1^ (Kajander et al., 2013). Thus, we lowered the rate constant of PP_i_ hydrolysis by a factor of 100 in our calculations (k_7_=5.7s^−1^; Supplementary Figure S2). The kinetic effect was basically the same: Nearly complete conversion (96%) of PP_i_ to P_i_ is reached at 20s (τ_1/2_=900ms). Hence, even very slow pyrophosphatases such as mPPases or vPPases can still drive amino acid activation by AARS enzymes to near completion. The reaction takes slightly longer than in the case of the type I sPPase, but is still complete in well under a minute. The reasons why the rate constants of the membrane bound PPases are so low is not known (Kajander et al., 2013), and it might be because their ion pumps work reversibly, synthesizing PP_i_ from 2P_i_ when the cations flow back through the membrane.

There are also type II sPPase (type II sPPase) that are much faster, and they hydrolyze PP_i_ with rate constants on the order of 1,700–3,000s^−1^ (Kajander et al., 2013). While all type I sPPases use Mg^2+^ ions to bind PP_i_ and water to negatively charged amino acids like aspartic acid and polarize water for PP_i_ hydrolysis by nucleophilic OH^−^ attack, type II sPPases additionally use Mn^2+^ (or Co^2+^) for binding and polarization, which probably relates to their higher rate constants. Type II sPPases have only been found in prokaryotes so far; they are common among clostridia and bacilli. Using the rate of 3,000s^−1^ for a type II sPPase (Kajander et al., 2013), we find that PP_i_ hydrolysis is 96% complete at 0.35s and 98% complete at 0.5s (τ_1/2_=36ms). The reaction rate of type II sPPase drives PP_i_ hydrolysis to completion in less than second, removing all PP_i_ substrate for the AARS back reaction. PPases I and II release the PP_i_ hydrolysis energy of −20 to −25kJ·mol^−1^ as heat in the cytosol. Hence, it is not pyrophosphatase thermodynamics, but its kinetics which drive the amino acid adenylation to high yield.

We point out that that in *E. coli* with, PP_i_ can accumulate to transient concentrations on the order of 1mM (Kukko and Heinonen, 1982) in exponentially growing cells, which would seem to create a conflict with the idea of a kinetic effect. Yet in natural environments, exponential growth is rarely if ever attained, as discussed in more detail in the next section. In the context of weighing kinetic vs. thermodynamic effects of PP_i_ production, we also recall the “uncomfortable” observation that deletion of *C. thermocellum* mPPase has no impact upon exponential growth (Holwerda et al., 2020), whereby chemostat cultures of *C. thermocellum*, which have high PP_i_ concentrations in the cytosol and a PP_i_-dependent glycolytic pathway, show clear signs of increased reversibility at the AARS reactions in the form of high concentrations of excreted amino acids (Holwerda et al., 2020). Whether such altruistic amino acid excretion into the environment *via* the AARS reaction would be manifested or sustainable in natural cellulose degrading environments over evolutionary timescales as opposed to chemostat growth conditions designed for biofuel yield is currently not known. It is also noteworthy that cells expend four ATP to generate a peptide bond even though one ATP would suffice, as peptide synthesis from aminoacyl phosphates (Kachalsky and Paecht, 1954) or non-ribosomal peptide synthesis (Martínez-Núñez and López y López, 2016) shows. The energetic difference between the one ATP required to form a peptide bond in solution vs. the four ATP that cells expend to make peptide bonds during translation can be seen as the energetic cost of structural information that is specified within a protein sequence (Haber and Anfinsen, 1962) plus the cost of its irreversible synthesis (Kornberg, 1962).

## Discussion

### Irreversibility in the Long-Term Evolution of Cells in Nature

What are the consequences of a PP_i_ irreversibility ratchet over geological timescales? From a physiological and energetic standpoint, the function of PP_i_ is always subordinate to ATP, because the source of the anhydride bond in PP_i_ in modern metabolism is always ATP, generated either *via* ion gradients or *via* substrate level phosphorylation. A critic might interject that thylakoid pyrophosphatases might be able to conserve energy as PP_i_ (Jiang et al., 1997), but if they do, it would be at the expense of one ATP per PP_i_ formed.

A critic might interject that Heinonen (2001) has summarized evidence to suggest that the measured cellular PP_i_ levels on the order of 1mM in logarithmically growing *E. coli* cells are too high to exert a kinetic effect of the kind that Kornberg had in mind. This issue can be illustrated with a passage from Kukko and Heinonen (1982), who measured the intracellular PP_i_ concentration of *E. coli* grown in batch culture with a doubling time of roughly 1h. They found that the PP_i_ concentration was constant at about 0.5mM during exponential growth. From this, they concluded that “*[…] the metabolic role of PP_i_ has been clouded by the widespread belief that PP_i_ formed in the metabolism is rapidly hydrolyzed in cells to inorganic phosphate and the concentration of PPi thus approaches zero in the cytoplasm. This view must be in error.*” We do not doubt their observations; their interpretation is the issue. Kukko and Heinonen (1982) cite several other papers where PP_i_ concentrations on the order of 0.1–2mM are reported, always from exponentially growing cells. Why are PP_i_ concentrations in exponentially growing cells misleading in the context of irreversibility?

When growth stops, so does PP_i_ production in the cytosol. But even after growth-dependent production of PP_i_ has ceased, PP_i_ continues to be hydrolyzed by pyrophosphatase activity. In a rare report, Danchin et al. (1984) measured PP_i_ after blocking ATP (hence PP_i_) synthesis in *E. coli*, and they found that PP_i_ levels dropped exponentially to 100μM within a minute and to 10μM within 10min, in line with our kinetic calculations (Supplementary Figure S2). Bielen et al. (2010) also noted that PP_i_ levels dropped when cells ceased exponential growth. Why do PP_i_ levels drop when growth is arrested? It is because PP_i_ is produced by growth processes (Klemme, 1976), but is hydrolyzed to phosphate by pyrophosphatases continuously, also in resting cells, independent of growth. This leads to a rapid drop in PP_i_ concentrations, which do in fact approach zero in the cytoplasm, once PP_i_ production is halted. This is why Danchin et al. (1984) observed a precipitous drop in PP_i_ concentrations once PP_i_ production was arrested.

The sources of PP_i_ in metabolism in typical cells (here, typical means cells that lack PP_i_-dependent glycolysis) have been known for decades. Klemme (1976) summarized the main sources of PP_i_ production in growing *E. coli* and the rates are which it is produced. In the units of μmol per 100mg biomass, the contributions to PP_i_ production in exponentially growing *E. coli* were: synthesis of protein (545), nucleic acids (67), polysaccharides (60), and lipids (60) for a total of 740. Using the fixed relationship between PP_i_ synthesis and growth in either rich or minimal medium, he was able to obtain good estimates for the rate of PP_i_ synthesis for several bacteria, which he set in relationship to the measured PPase activity for the same bacteria, allowing him to calculate the ratio of rates (μmoles·h^−1^·mg protein^−1^) for PP_i_ production and PP_i_ hydrolysis. For eight exponentially growing bacterial species (six Gram negative, two Gram positive), he found that the ratio of PP_i_ hydrolysis to PP_i_ synthesis was 79, 73, 57, 33, 14, 10, 8, and 1 (Table II of Klemme, 1976). The average value was 35; the value for *E. coli* was 14. That is, the rate of *E. coli* PP_i_ hydrolysis is 14 times higher than the rate of PP_i_ production from growth processes. Even if the ratio is only 1, when exponential growth is arrested, pyrophosphatase activity remains, which will relentlessly gnaw away at PP_i_ concentrations until they essentially reach zero or until rapid growth is resumed, such that rates of cytosolic PP_i_ production can exceed PP_i_ hydrolysis. A number of microbes possess Mn^2+^- or Co^2+^-dependent sPPases, called family II sPPases, that have a catalytic rate higher than that of typical sPPases (Kajander et al., 2013).

The foregoing raises two important points. The first is that PP_i_ levels in exponentially growing cells are not a good proxy for the function of PP_i_ over evolutionary time. This is because sustained exponential growth is never attained for microbes in the environment or during evolution, as they are mainly starved for nutrients in the wild. In the largest microbial community known, marine sediment, cells do not actually grow, they just slowly die as organic nutrients become limiting (Orsi et al., 2020). In such environments, the standard concept of doubling times does not apply to growth or survival, as cell mass never doubles, it just turns over from one living cell to another, with turnover times on the order of tens to thousands of years (Hoehler and Jørgensen, 2013). In starved cells as they exist in sediment, ATP synthesis is orders of magnitude slower than in exponentially growing cells and PP_i_ production is governed by the rate of protein synthesis, meaning that on time scales of days, months, and years, trace pyrophosphatase activity will hold the cytosolic PP_i_ concentration close to zero, even if the enzyme’s affinity for PP_i_ is comparatively low. However, measured values of *K*_m_ (the substrate concentration at half maximal enzymatic reaction rate) for PP_i_ for sPPases are not high, and they tend to be in the range of 1μM to 1mM in *E. coli* (Supplementary Table S3) and values of catalytic rate tend to be on the order of 200–400s^−1^ (Avaeva, 2000; Kajander et al., 2013), meaning that over long time scales, PPases keep PP_i_ levels in cells too low to permit the enzymatic reactions of translation or nucleic acid polymerization from running backwards, especially at the extremely slow PP_i_ production rates of starved microbial communities.

We say “extremely slow PP_i_ production rates.” How slow is slow? We can provide an estimate. Starved cells are small. An exponentially growing *E. coli* cell has about 2 million proteins with on average 300 amino acids each (Milo et al., 2010). If a starved cell is half that size, roughly 300 million peptide bonds are required for its formation. There are 32 million seconds in a year, such that if the turnover time of starved cell is on the order of 10years, one peptide bond per second is formed, on average, during the formation of the cell. That might not sound too slow, because a ribosome can form about 10 peptide bonds per second. But a small *E. coli* cell has on the order of 10,000 ribosomes, such that in a starved cell with a 10-year turnover time, an individual ribosome might perform an elongation step on the order of roughly once every 3h or 10^−4^ peptide bonds per second. In terms of molecular processes that are extremely slow, the rate of PP_i_ production is 100,000 times slower than from translation during exponential growth. This example underscores the value to the cell of translation (aminoacyl tRNA synthesis) being an irreversible process over evolutionary timescales, and the essential function that irreversibility plays by acting as a ratchet’s pawl to prohibit the back reaction of aminoacyl tRNA formation, quantitatively the most important energetic expense a cell encounters (Figure 1).

A second important point concerns (rare) examples in which sPPases are lacking in the genome such that PP_i_ reaches high cytosolic concentrations rendering translation theoretically reversible. This can occur in cells that have PP_i_-based glycolysis, such as *C. thermocellum*, where ion-pumping membrane-bound PPases (mPPases) are present (Holwerda et al., 2020). In such cells, which are sugar specialists, PP_i_ levels can exceed 20mM during chemostat growth, whereby the metabolic source of such high PP_i_ levels is still unclear and deletion of the *C. thermocellum* mPPase has no impact upon growth (Holwerda et al., 2020). The existence of cells with PP_i_-dependent carbon metabolism lacking high activities of sPPases in chemostat growth on very rich medium do not invalidate Kornberg’s principle of irreversibility over evolutionary timescales. Rather they constitute an evolutionarily derived special case of adaptation to growth on sugar. As outlined in the introduction, PP_i_ utilizing glycolytic enzymes tend to occur among microbes that have specialized to sugar-rich environments, including human parasites such as *Entamoeba*, *Giardia*, and trypanosomes, or cellulose- and saccharose-degrading bacteria and archaea, but also in plants with their specialized sugar synthesizing compartment, the plastid. Proton-pumping mPPases are often associated with acidocalcisomes, membrane-bounded compartments that occur in some prokaryotes and in some eukaryotes including parasites and plants (Docampo et al., 2005). The functions discussed for acidocalcisomes include among other things storage of cations, phosphate and polyphosphate, calcium signaling, and osmoregulation but no evidence for an involvement in energy metabolism of acidocalcisomes or their mPPase has emerged so far (Docampo and Huang, 2016).

In the bigger picture of microbial evolution, sugar-dependent lifestyles cannot be ancestral, and they have to be derived. The early earth was barren and offered CO_2_, not glucose, as the main environmental carbon source (Schönheit et al., 2016). In the modern crust (Smith et al., 2019) and marine sediments (Orsi et al., 2020), where most cells on Earth have always resided (McMahon and Parnell, 2018), net growth is almost non-existent due to nutrient limitations (Orsi et al., 2020). Particularly in low-energy environments, PP_i_ irreversibility at the quantitatively dominant (in terms of PP_i_ synthesis) AARS reaction acts like a ratchet’s pawl that keeps aminoacyl tRNAs moving in solely in the direction of translation, even if translation is slow for reasons of substrate limitation or prolonged starvation.

## Conclusion

### Why Nature Chose Triphosphates

The role of PP_i_ in evolution raises a question similar to Westheimer’s “why phosphate,” namely “why triphosphates?” Westheimer (1987) proposed that phosphates became energy carriers because of the metastability of the various bonds that phosphate forms with organic compounds under physiological conditions. By examining the role of PP_i_ in the core and in the central dogma, we found that the central function of PP_i_ producing reactions is not that of an energy currency in any case. In metabolism, PP_i_ is always generated from nucleoside triphosphates. This is also true for mPPases, where the ion gradients that are required for PP_i_ synthesis are generated at ATP expense. Formulated directly, we find no evidence that PP_i_ served as a primordial energy currency or that it serves as an energy currency today. Rather, it appears that the role of PP_i_ is to impart direction upon the most essential operations of life: biosynthesis of cofactors, the biosynthesis of the monomeric building blocks of proteins and nucleic acids, and the polymerization of those building blocks into the catalysts and information carriers of cells (Figure 1).

**Figure 2:**
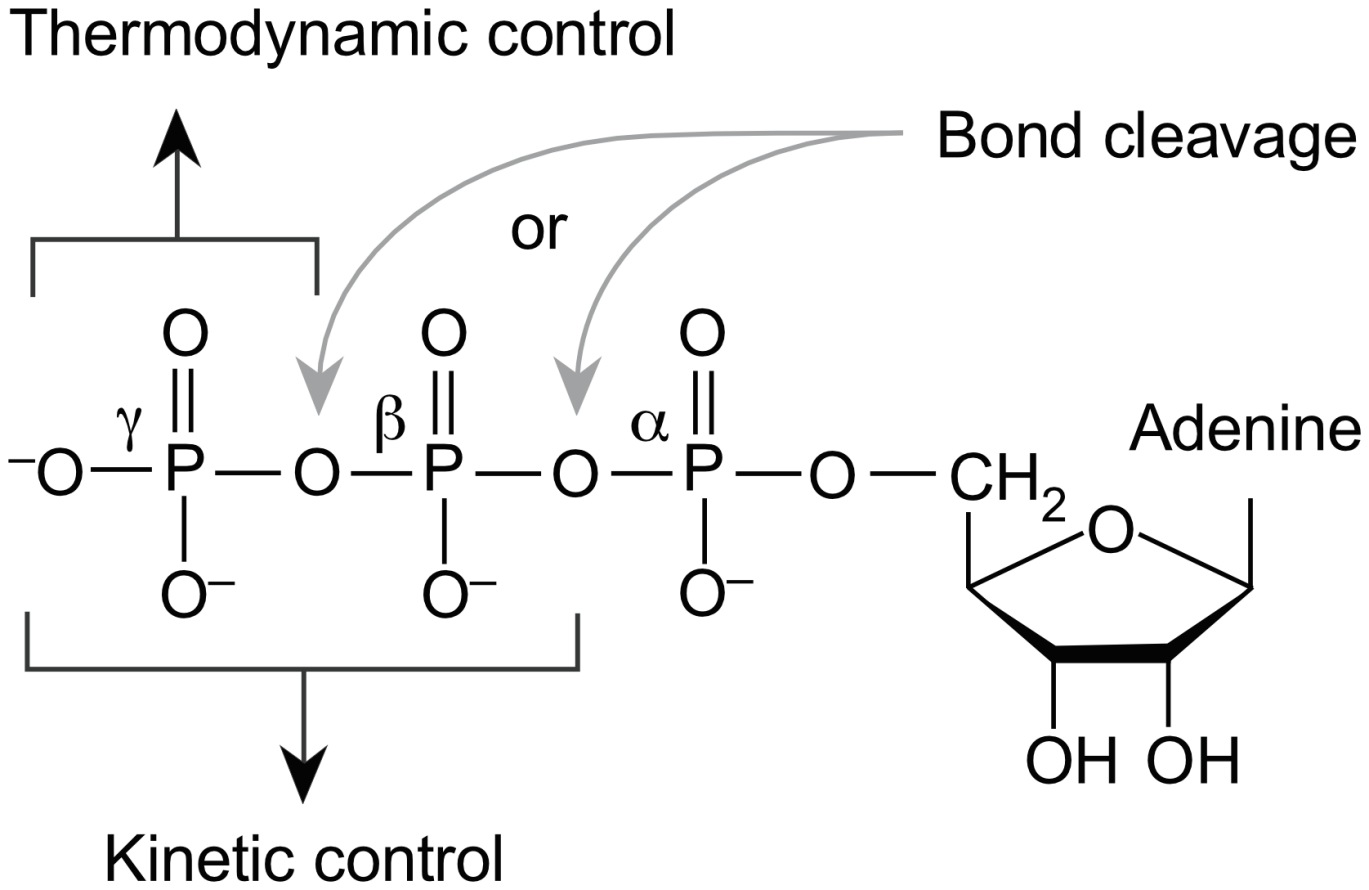
Role of nucleoside triphosphates in metabolic evolution. Coupling of ATP hydrolysis to P_i_ and ADP or to phosphorylation releases −32kJ·mol^−1^ under standard physiological conditions, shifting the equilibrium of otherwise mildly endergonic reactions in the direction of product formation. Such reactions are under thermodynamic control, the products are more stable than the educts, but such reactions are usually reversible under physiological conditions of the cell, unless ∆*G* is very large. Coupling of ATP hydrolysis to PP_i_ and AMP or to adenylation releases −45kJ·mol^−1^ under standard physiological conditions, similar to the free energy change for acyl phosphate hydrolysis, but the ubiquitous presence of pyrophosphatase activity in cells leads to immediate PP_i_ hydrolysis, such that a product for the reverse reaction is removed. Even if the reverse reaction was thermodynamically favorable, it cannot take place because an educt (PP_i_) is lacking, making the reverse reaction orders of magnitude slower than the forward reaction, placing it under kinetic control.

Why did nature specifically choose nucleoside triphosphates as the universal energy currency? That is a fundamentally different question from why nature chose phosphate (Westheimer, 1987; Liu et al., 2019) because many biological compounds harbor phosphate bonds with a large free energy of hydrolysis (Decker et al., 1970), but only triphosphates are the universal energy currency in all lineages today. Irreversibility provides the answer. Triphosphates can generate either P_i_ or PP_i_. This subtle property reveals why triphosphates became the universal energy currency in cells and why they have not been replaced in 4 billion years of evolution (Figure 2). How so?

Nucleoside triphosphates such as ATP have two phosphoanhydride bonds. The β-phosphate in ATP can be cleaved on either side (Figure 2). ATP-dependent enzymatic reactions that release P_i_ utilize ATP as a currency of thermodynamic control, making ∆*G* of the reaction sufficiently negative (or the net activation energy sufficiently low) to allow the reaction to go forward. ATP-dependent reactions that release PP_i_ also have a thermodynamic component, but the irreversibility of the reaction conferred by PP_i_ hydrolysis under physiological conditions places the reaction under kinetic rather than thermodynamic control.

No biochemical energy currency other than (nucleoside) triphosphates offers, within the same compound, the alternative of exerting either thermodynamic or kinetic control over a reaction. This property is specific to triphosphates. It allowed primordial enzymes to exert either kinetic control or thermodynamic control over catalyzed reactions, depending upon which anhydride bond of the β-phosphate was cleaved. This in turn imparted the option of evolutionary refinement of an initial catalytic activity among ATP utilizing enzymes according to the prevailing selective forces in a given cellular environment. An early onset of PP_i_-dependent irreversibility in metabolism would not require the presence of a pre-existing inorganic pyrophosphatase enzyme activity at the site of origins, because the reaction can be catalyzed by inorganic ions alone, such as Mg^2+^ (Stockbridge and Wolfenden, 2011), which catalyzes hydrolysis in the active site of many modern pyrophosphatase enzymes (Varfolomeev and Gurevich, 2001).

This, in turn, is the reason why nucleoside triphosphates became fixed in both monomer and polymer biosynthesis in the metabolism of LUCA and have not been displaced since. From an ancestral state in which acyl phosphates provided thermodynamic impetus and a means of energetic coupling in enzymatic reactions, the advent of nucleoside triphosphates changed the nature of early biochemical evolution by introducing the option of kinetic irreversibility. Triphosphates offered primordial enzymes a means to exert either kinetic control or thermodynamic control over catalyzed reactions with one and the same energy currency. The only evident alternative solutions would have been (i) to maintain two distinct energetic currencies in the cell, one for energetic and one for kinetic purposes (an event for which there is no evidence) or (ii) to abandon one of the functions (which is not a viable option over evolutionary time). The ability of triphosphates to function in roughly 2/3 of phosphoanhydride bond expenditure as a currency of energy (thermodynamic drive) and in roughly 1/3 of phosphoanhydride bond expenditure as a currency of irreversibility (kinetic drive; Figure 1) is the reason they became – and remained – life’s universal energy currency.

## Data Availability Statement

The original contributions presented in the study are included in the article/Supplementary Material; further inquiries can be directed to the corresponding author/s.

## Author Contributions

JW collected and analyzed data, participated in project design, and revised the manuscript. KK performed the kinetic calculations and contributed in data interpretation. WM wrote the first manuscript draft, performed literature research, visualization, and conceived and supervised the study. All authors contributed to the article and approved the submitted version.

## Funding

This work was supported by the European Research Council (Advanced Grants eMicrobevol and EcolMetabOrigin to WM), the Deutsche Forschungsgemeinschaft (Ma 1426/21-1 to WM), and the Volkswagen Foundation (VW 96742 to WM).

## Conflict of Interest

The authors declare that the research was conducted in the absence of any commercial or financial relationships that could be construed as a potential conflict of interest.

## Publisher’s Note

All claims expressed in this article are solely those of the authors and do not necessarily represent those of their affiliated organizations, or those of the publisher, the editors and the reviewers. Any product that may be evaluated in this article, or claim that may be made by its manufacturer, is not guaranteed or endorsed by the publisher.
