# Supplementary material for: Pyrophosphate and Irreversibility in Evolution, or why PPi Is Not an Energy Currency and why Nature Chose Triphosphates
Source: Front Microbiol. 2021 Oct 6;12:759359. doi: 10.3389/fmicb.2021.759359 (PMC8575175; doi:10.3389/fmicb.2021.759359)
Supplement: Supplementary file 4 [file Data_Sheet_1.PDF]

Supplementary Material

Panel **A** of **Supplementary Figure 1** shows the kinetic scheme taken from Pope et al. (1998) with hydrolysis of PP_i added. In the first step, the amino acid AA or the activator ATP attaches to the enzyme E followed by addition of the second reactant to form the E•AA•ATP complex. This complex reacts to the adenylated amino acid AA-AMP with pyrophosphate PP_i and the enzyme E noncovalently attached (E•AA-AMP• PP_i). Then PP_i leaves the complex and the product E•AA-AMP is formed with free PP_i ready to be hydrolyzed by pyrophosphatase to two inorganic phosphate ions P_i. Panel **B** shows the rate equations used to obtain the concentration versus time profiles by numerical integration with Mathcad (Mathcad 2001; Mathsoft Engineering & Education, Inc.). The experimental rate constants were obtained from Pope et al., 1998. Initial concentrations of 1 mM amino acid, enzyme and ATP were used, and integration was carried out up to 20 s.

Panel **A** of **Supplementary Figure 2** shows nearly complete (96 %) conversion of the amino acid (in this case isoleucine) to the adenylated amino acid (blue curve) in the presence of pyrophosphatase (k_7 = 570 s^{-1}) after 0.5 s. The experimental PPase rate constant was obtained from Stockbridge and Wolfenden (2011). In panel **B**, the same reactions are shown for the absence of pyrophosphatase (k_7 = 0 s^{-1}). Adenylated isoleucine rapidly increases up to 0.19 mM (half-life $\tau_{1/2}$ = 9 ms) and stays stationary at that value up to calculated 0.5 s reaction time. The stationary concentration of 0.19 mM reflects the fast approach to equilibrium with low product concentration in the absence of pyrophosphatase. Pyrophosphatase activity (panel **A**) removes PP_i from this equilibrium and therefore gradually shifts the equilibrium to concentrations with more product formed (half-life $\tau_{1/2}$ = 41 ms) and finally (0.5 s) to nearly complete product formation.

As shown above, PP_i hydrolysis as a driving force for nearly complete adenylation is remarkably stable concerning variation of the PPase rate constant k_7. Similar stability is observed for other rate constants of amino acid adenylation of isoleucine. For reactions 2, 3 and 6, equilibrium constants were obtained experimentally by Pope et al. (1998). To obtain the rate constants we assumed substrate binding rate constants k_{+2}, k_{+3} and $k_{-6} = 10^6$ $M^{-1}s^{-1}$ similar to the experimental binding rate constant of the isoleucine substrate to the enzyme k_{+1}. Then their reverse rate constants were calculated such that the equilibrium constants had the experimental values. We varied the substrate binding rate constants in a large range to investigate their influence on the product yield. For example, with a rate constant $k_{-6} = 10^7$ $M^{-1}s^{-1}$ for the reverse reaction of PP_i with E•AA-AMP, we obtain 96 % conversion at 0.4 s. Similar high conversion yields were received for $k_{-6} = 10^8$ to 10^9 $M^{-1}s^{-1}$ with the upper value approaching diffusion limited rate constants in a more viscous solvent than water. Of course, k_{+6} had to be increased as well to get the experimental $K_6 = k_{+6}/k_{-6}$ = 250 µM. The reaction times were different for the different rate constants, but high product yields were obtained in any case if k_7 = 570 s^{-1} is used.

Panel **C** of **Supplementary Figure 2** shows the concentration profiles at short times in more detail. As expected, the concentrations of the enzyme E_t and the substrates AA_t and ATP_t decrease with time t. The complex E•AA_t is an intermediate and appears at larger concentrations than the intermediate E•ATP_t (because the equilibrium constant for E•ATP_t decay K_2= 2.5 mM is large). E•AA•ATP_t is produced from E•AA_t and E•ATP_t and therefore appears with some delay relative to the former. The adenylated products, PP_i and P_i appear still later and have low concentrations in the time range up to

5 ms. The influence of the pyrophosphatase can already be seen here however, because the PP_i concentration (red dashed curve) is below the E•AA•AMP product concentration (blue curve).

A

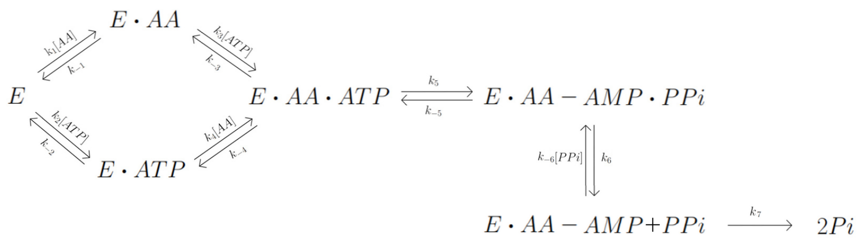

B

$$\frac{dE\cdot AA}{dt} = k_1 \times E \times AA - k_{-1} \times E\cdot AA - k_3 \times E\cdot AA \times ATP + k_{-3} \times E\cdot AA\cdot ATP \quad (1)$$

$$\frac{dE}{dt} = -k_1 \times E \times AA + k_{-1} \times E\cdot AA - k_2 \times E \times ATP + k_{-2} \times E\cdot ATP \quad (2)$$

$$\frac{dATP}{dt} = -k_2 \times E \times ATP + k_{-2} \times E\cdot ATP + k_{-3} \times E\cdot AA\cdot ATP - k_3 \times E\cdot AA \times ATP \quad (3)$$

$$\frac{dE\cdot ATP}{dt} = -k_2 \times E \times ATP + k_{-2} \times E\cdot ATP - k_4 \times E\cdot ATP \times AA + k_{-4} \times E\cdot AA\cdot ATP \quad (4)$$

$$\frac{dE\cdot AA\cdot ATP}{dt} = k_4 \times E\cdot ATP \times AA - k_{-4} \times E\cdot AA\cdot ATP + k_3 \times E\cdot AA \times ATP - k_{-3} \times E\cdot AA\cdot ATP - k_5 \times E\cdot AA\cdot ATP + k_{-5} \times E\cdot AA{-}AMP\cdot PPi \quad (5)$$

$$\frac{dE\cdot AA{-}AMP\cdot PPi}{dt} = k_5 \times E\cdot AA\cdot ATP - k_{-5} \times E\cdot AA{-}AMP\cdot PPi - k_6 \times E\cdot AA{-}AMP\cdot PPi + k_{-6} \times E\cdot AA{-}AMP \times PPi \quad (6)$$

$$\frac{dE\cdot AA - AMP}{dt} = k_6 \times E\cdot AA{-}AMP\cdot PPi - k_{-6} \times E\cdot AA{-}AMP \times PPi \quad (7)$$

$$\frac{dPPi}{dt} = -k_7 \times PPi + k_6 \times E\cdot AA{-}AMP\cdot PPi - k_{-6} \times E\cdot AA{-}AMP \times PPi \quad (8)$$

$$\frac{dAA}{dt} = -k_1 \times E \times AA - k_4 \times E\cdot ATP \times AA + k_{-1} \times E\cdot AA + k_{-4} \times E\cdot AA\cdot ATP \quad (9)$$

$$\frac{dPi}{dt} = +2 \times k_7 \times PPi \quad (10)$$

Supplementary Figure 1. The kinetic effect of PP_i in translation. **A** Kinetic scheme of substrate binding and activation of isoleucine by adenylation in isoleucyl-tRNA synthetase of *Staphylococcus aureus*. Reactants and products: E (AARS enzyme), AA (amino acid), ATP

(adenosine triphosphate), AMP (adenosine monophosphate), PP_i (pyrophosphate), P_i (inorganic phosphate). Symbols: • noncovalent binding, – covalent binding, k_i rate constants. Modified from Pope et al., 1998. **B** Kinetic equations for adenylation of amino acids in AARS (Aminoacyl-tRNA-Synthetase). Concentrations: E (AARS enzyme), AA (amino acid), ATP (adenosine triphosphate), AMP (adenosine monophosphate), PPi (pyrophosphate), P_i (inorganic phosphate). Symbols as in A, x multiplication. Rate constants and equilibrium constants $k_{+1} = 2.2 \times 10^6\ M^{-1}s^{-1}$, $k_{-1} = 130\ s^{-1}$, $K_2 = k_{-2}/k_{+2} = 2.5$ mM, $K_3 = k_{-3}/k_{+3} = 70$ µM, $K_4 = 1.7 \times 10^6\ M^{-1}s^{-1}$, $k_{-4} = 3\ s^{-1}$, $K_5 = 60\ s^{-1}$, $k_{-5} = 170\ s^{-1}$, $K_6 = k_{+6}/k_{-6} = 250$ µM were obtained from the experiments of Pope et al. (1998). The rate constant for hydrolysis of inorganic pyrophosphate by *Escherichia coli* inorganic pyrophosphatase $K_7 = 570\ s^{-1}$ is obtained from Stockbridge and Wolfenden (2011).

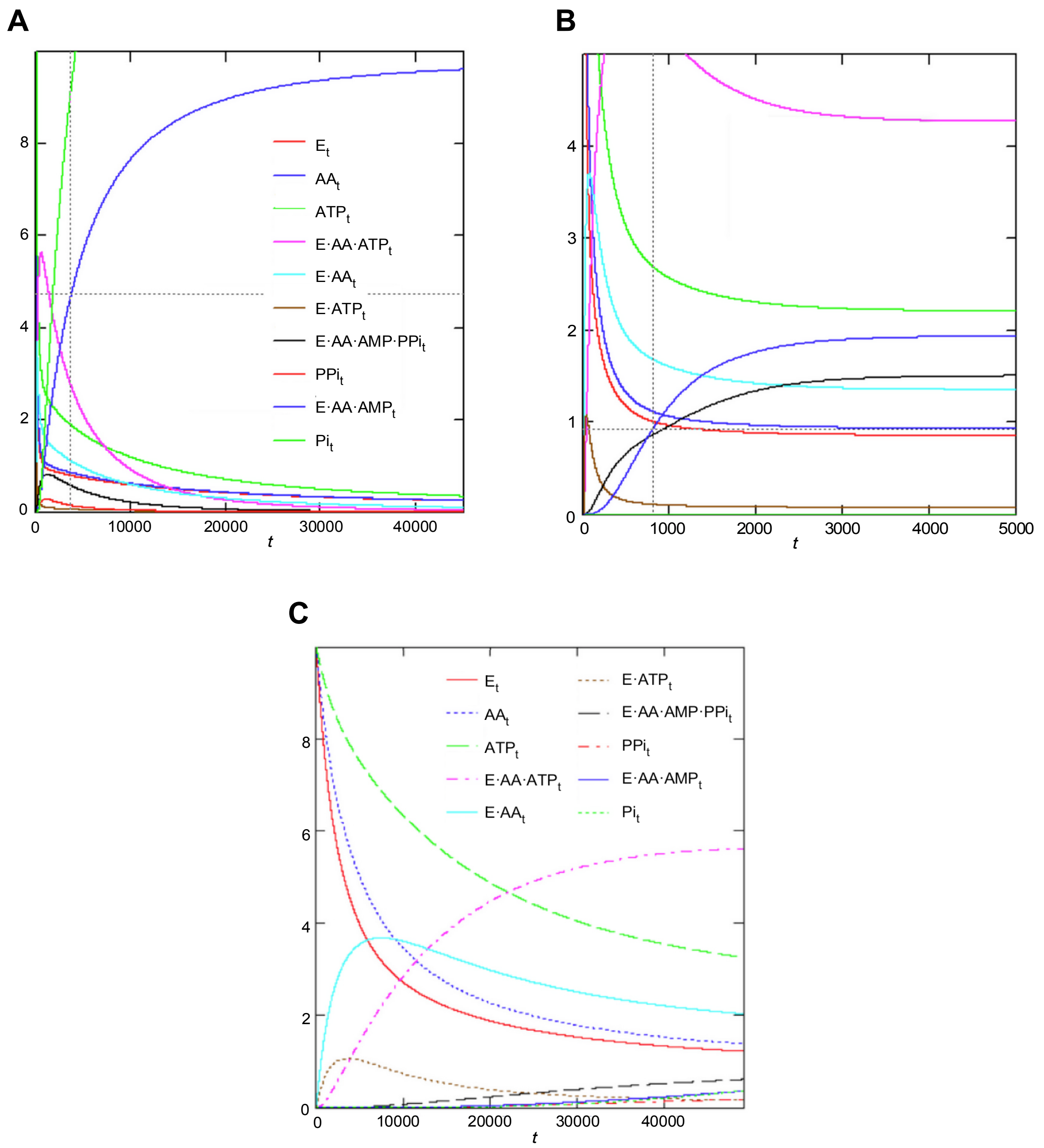

Supplementary Figure 2. Time dependence of reactant and product concentrations in isoleucyl-tRNA synthetase **A** with inorganic pyrophosphatase of *E. coli* (k_7 = 570 s^{-1}). The product (adenylated isoleucine-enzyme complex: E•AA-AMP; blue curve) increases to 0.96 mM for 1 mM initial isoleucine concentration at 0.5 s reaction time ($4.5 \cdot 10^4$ time steps/($9 \cdot 10^4$) s time increment) with a half-life period of 41 ms (0.48 mM half-concentration at 3711/($9 \cdot 10^4$) s = 41 ms) as indicated by the vertical dashed line. **B** Time dependence of reactant and product concentrations in isoleucyl-tRNA synthetase without pyrophosphatase (k_7 = 0 s^{-1}). The product (adenylated isoleucine-enzyme complex: E•AA-AMP; blue curve; curves labelled as in A increases to stationary 0.19 mM for 1

mM initial isoleucine concentration at 0.5 s reaction time with a half-life period of 9 ms (0.095 mM half-concentration at $817/(9\cdot10^4)$ s = 9.1 ms) as indicated by the vertical dashed line. **C** Initial time dependence (0 – 4.9 ms; $4.9\cdot10^4/(1\cdot10^7)$ s) of reactant and product concentrations in isoleucyl-tRNA synthetase with PPi hydrolysis by pyrophosphatase (k_7 = 570 s^{-1}). Mass balance was checked and fulfilled for every reaction time, that is, the sum of all concentrations containing a reaction component must be equal to the initial concentration of that component.
